# Pneumonia and Steam-Coils

**Published:** 1875-03

**Authors:** 


### PNEUMONIA AND STEAM-COILS.

Pneumonia has been unusually rife and fatal this winter. It is a disease to which all ages are subject, but it is much more dangerous in adults than in children. Among grown people only one in ten who are stricken with this disease, recover. It destroys life very speedily, as it attacks the very citadel, the breathing organs. Some of the causes which induce it, as well as some of its peculiarities, may be learned by a perusal of our article, entitled, "The Most Deadly Disease." The breathing of vitiated air, and sudden transitions from extreme heat to extreme cold, as well as inspiration through the mouth and not through the nose, tend to cause it.

Persons who cut their throats for the purpose of committing suicide, are likely to die from this disease, as the air rushes in through the orifice in the severed windpipe and the unstrained and unwarmed air speedily induces it.

Steam-heating devices, which supply no new air, but really heat up, over and over again, the dead air long ago exhausted of its vitality, so debilitate the system as to render it impossible to resist an attack of this fell disease. After breathing this hot, bad air, for several hours, the lungs are scarcely able to endure with safety the transition to the cold pure air without.

The fact is, we shall have to abandon these steam-coils sooner or later, unless we can supplement them in all cases with some perfect system, by which new air can be constantly introduced. It would be far better if the air could be first heated by the coils and then discharged into the room, as this would secure constant change. Until this is done, we are wise to adhere to the best hot-air furnace to be had, and to force such great quantities of fresh air, well heated, into the room as to permit us to have a window constantly open for purposes of ventilation. This is entirely feasible, as we can testify. We have spent nearly all the hours of the twenty-four throughout this entire winter in a room heated by a register from such a furnace, and with one or more windows constantly open, to the extent of several inches. An open window has been necessary for comfort, as the mercury would invariably ascend to 90° if the window was closed.

We intended to speak of the furnace used, but we lack space. It is the kind of which we have spoken before, the "Gothic," of Mr. A. M. Lesley, 224 West Twenty-Third Street, New York. It is a wonderful heater, a fact fully attested by the following letter from the editor of the *Cultivator and Country Gentleman*, just at hand:

UNION SPRINGS, N. Y.,
*February* 5, 1875.

*Dear Sir:* Since I have had two additional furnaces set up in buildings under my care this winter, I am more than ever satisfied with the superiority of the Gothic Furnace. There are two prominent points: first, great simplicity; and second, nearness of heating to the burning coal, causing it to throw out heat more strongly. The latter is an important advantage. There are other advantages, but these have particularly attracted my attention of late. Cheapness, ease of management, *purity of air*, durability, &c., are not to be overlooked. The complex base-burners are proving unsatisfactory. J. J. THOMAS,
*Editor of Cultivator and Country Gentleman.*

